# Clinically Approved Drugs Inhibit the *Staphylococcus aureus* Multidrug NorA Efflux Pump and Reduce Biofilm Formation

**DOI:** 10.3389/fmicb.2019.02762

**Published:** 2019-12-03

**Authors:** Saskia Zimmermann, Mareike Klinger-Strobel, Jürgen A. Bohnert, Sindy Wendler, Jürgen Rödel, Mathias W. Pletz, Bettina Löffler, Lorena Tuchscherr

**Affiliations:** ^1^Institute of Medical Microbiology, Jena University Hospital, Jena, Germany; ^2^Center for Sepsis Control and Care, Jena University Hospital, Jena, Germany; ^3^Institute of Infectious Diseases and Infection Control, Jena University Hospital, Jena, Germany; ^4^Institute of Medical Microbiology, Greifswald University Hospital, Greifswald, Germany

**Keywords:** multidrug resistance, biofilm, NorA, *S. aureus*, clinical drugs

## Abstract

*Staphylococcus aureus* has acquired resistance to antibiotics since their first use. The *S. aureus* protein NorA, an efflux pump belonging to the major facilitator superfamily (MFS), contributes to resistance to fluoroquinolones (e.g., ciprofloxacin), biocides, dyes, quaternary ammonium compounds, and antiseptics. Different compounds have been identified as potential efflux pump inhibitors (EPIs) of NorA that result in increased intracellular concentration of antibiotics, restoring their antibacterial activity and cell susceptibility. However, none of the currently known EPIs have been approved for clinical use, probably due to their toxicity profiles. In the present study, we screened approved drugs for possible efflux pump inhibition. By screening a compound library of approximately 1200 different drugs, we identified nilotinib, a tyrosine kinase inhibitor, as showing the best efflux pump inhibitory activity, with a fractional inhibitory concentration index of 0.1875, indicating synergism with ciprofloxacin, and a minimum effective concentration as low as 0.195 μM. Moreover, at 0.39 μM, nilotinib, in combination with 8 μg/mL of ciprofloxacin, led to a significant reduction in biofilm formation and preformed mature biofilms. This is the first description of an approved drug that can be used as an efflux pump inhibitor and to reduce biofilms formation at clinically achievable concentrations.

## Introduction

*Staphylococcus aureus* (*S. aureus*) is a widespread opportunistic pathogenic organism that can cause a wide range of illnesses, including skin infections, endocarditis, osteomyelitis, and sepsis with mild to life-threatening outcome (Lowy, 1998; Tuchscherr and Loffler, 2016). The problem is exacerbated by increasing antibiotic resistance among *S. aureus* clinical isolates, such as methicillin resistant strains (MRSA). MRSA strains appear to possess the ability to constantly acquire additional antibiotic resistance genes (Shorr, 2007; Felicetti et al., 2017). This ability of *S. aureus* is not only limited to planktonic bacteria but also extends to biofilms. Biofilms are matrix-encased communities formed by bacteria on surfaces, leading to higher antibiotic tolerance, thus enabling higher persistence levels (Suresh et al., 2019). The treatment of biofilm is further complicated by the range of bacterial phenotypic variants appearing in a fully formed biofilm (Yarwood et al., 2007).

Active efflux is considered to be the first-line of defense for bacteria against antimicrobials (Sundaramoorthy et al., 2018). Efflux is the extrusion of a substrate out of a bacterial cell. Efflux pumps can be encoded chromosomally and on a plasmid. To date, there are known more than 30 efflux pump genes alone in *S. aureus* (Bhaskar et al., 2016). NorA is the most studied pump in *S. aureus* (Buonerba et al., 2017). The *norA* gene encodes for a 42 kDa protein in the bacterial cell membrane (Wang et al., 2018). This protein is encoded chromosomally and expressed on a basal level (Kaatz and Seo, 1995; Costa et al., 2013). There are two ways that may lead to an overproduction of the NorA efflux pump, by mutations in the *norA*-encoding gene and by the inducible expression of *norA* via regulatory genes (Costa et al., 2013). The substrate range of the NorA efflux pump is broad, including fluoroquinolones like ciprofloxacin, biocides, dyes, quaternary ammonium compounds and antiseptics (Fontaine et al., 2014; Bhaskar et al., 2016). In 43% of *S. aureus* strains the *norA* gene is overexpressed, particularly in MRSA strains (Fontaine et al., 2014; Astolfi et al., 2017). Overexpression of the *norA*-gene is associated with increased resistance toward the NorA substrates, leading to a diverse resistance pattern including fluoroquinolone resistance (Kaatz and Seo, 1995). Moreover, efflux pumps from different microorganisms have been linked to virulence and biofilm formation (Piddock, 2006; Kvist et al., 2008; Baugh et al., 2012; Lopes et al., 2017; Wang-Kan et al., 2017). Efflux pumps play a role in biofilm formation by (i) excretion of extracellular matrix molecules, (ii) excretion of quorum sensing molecules that coordinate biofilm formation, (iii) efflux of harmful molecules and (iv) influencing surface adhesion (Alav et al., 2018). Efflux pump gene expression is up-regulated in *S. aureus* during biofilm growth (He and Ahn, 2011). Furthermore, several studies have shown that previously identified efflux pump inhibitors (EPIs) significantly decrease biofilm formation in *E. coli*, *Pseudomonas aeruginosa*, *Pseudomonas putida*, *S. aureus*, and *Klebsiella pneumonia* (Ikonomidis et al., 2008; Kvist et al., 2008; Baugh et al., 2014). Developing inhibitors of the NorA efflux pump is a promising approach to potentially not only reverse multidrug resistance (MDR) but also inhibit biofilm formation and virulence (Kalia et al., 2012; Singh et al., 2017; Abd El-Baky et al., 2019). However, to date no clinically approved drug has been identified that could be administered as an EPI. Therefore, we set out to screen already clinically approved drugs for possible inhibitory effects on the NorA pump.

## Results

### Molecular Docking, Virtual Screening, and MIC

Because the crystal structure of NorA is currently unknown (Astolfi et al., 2017; Felicetti et al., 2017), we used the EmrD efflux pump protein (like NorA, EmrD is a member of the major facilitator superfamily, an efflux pump family) as a template for a homology model to identify possible efflux pump inhibitors (EPIs). EmrD, identified in *E. coli*, is a member of the MFS extensively disseminated among the Gram-positive and -negative bacteria (Yin et al., 2006). The crystal structure of the EmrD-efflux pump is well known and it presents a high homology to the NorA efflux pump (with 19% identity and 41% similarity) (Yin et al., 2006; Law et al., 2008; Yan, 2013). Due to this homology, the EmrD is the most common model used for investigating EPIs for NorA (Thai et al., 2015). The compound library was docked against the entire surface of the NorA homology model and the free energies of binding of the best binding poses were calculated (Table 1). For further experiments, the four substances with the highest estimated binding affinities were chosen as well as arbitrarily one additional result from the ten best hits, one from the twenty best hits, two from the 100 best hits, one from the 500 best hits, and one from the 1000 best hits (Table 1). The results showed that substances with the highest and lowest binding affinities were dihydroergotamine (binding energy of −13.2 kcal/mol) and naftifine (binding energy of −8.4 kcal/mol), respectively. Reserpine, an established NorA efflux pump inhibitor (Fontaine et al., 2015), was chosen as a control for further experiments (binding energy -9.4 kcal/mol).

**TABLE 1:** Screening of potential inhibitors of NorA.

**Ligand**	**Binding energy in kcal/mol**
Dihydroergotamine	−13.2
Ergoloid	−12.4
Pimozide	−11.8
Telmisartan	−11.8
Maraviroc	−11.6
Azelastine	−11.3
Ketoconazole	−10.9
Nilotinib	−10.8
Doxazosin	−9.4
Naftifine	−8.4
Reserpine	−9.4

**Binding energies of candidate compounds docked to active site residues of the NorA homology model were calculated using AutoDock Vina.**

Next, the minimal inhibitory concentration (MIC) of ciprofloxacin and compounds selected by molecular docking was investigated for the strains SA1199 (wild type) and SA1199B (NorA overproduced, *norA*+++.) (Table 2). The MIC value for ciprofloxacin was higher for SA1199B than SA1199, whereas the other compounds showed similar values for both strains. A broad range of MICs for the investigated compounds toward *S. aureus* SA1199B (*norA*+++) were observed, from 12.5 μM (pimozide) up to 800 μM or above for maraviroc, nilotinib, naftifine, and reserpine (Table 2). Taken together, our results indicate that pimozide, maraviroc, nilotinib and naftifine are potential EPIs of NorA.

**TABLE 2:** MICs of the tested compounds (in μM unless otherwise indicated).

**Compounds**	**MIC SA1199**	**MIC SA1199B** **(*norA*+++)**
Ciprofloxacin	0.25 μg/mL	4 μg/mL
Dihydroergotamine	200	200
Ergoloid (Dihydroergotoxinemesylate)	100	100
Pimozide	12.5	12.5
Telmisartan	400	400
Ketoconazole	50	50
Maraviroc	>800	>800
Azelastine	400	400
Nilotinib	800	800
Doxazosin	200	200
Naftifine	>800	>800
Reserpine	800	800

**The concentrations are the results of three independent experiments.**

### Synergy Assays

The combination of ciprofloxacin and the compounds was tested with SA1199B to evaluate possible synergistic activity. A fixed amount of compound (1/4 of the MIC but not higher than 100 μM as higher concentrations were considered unachievable in the clinic because of toxicity) was added to different amounts of ciprofloxacin to determine an effect. The reduced concentration of ciprofloxacin needed to inhibit bacterial growth is shown in Table 3. According to the fractional inhibitory concentration index (FICI) values, pimozide, ketoconazole, maraviroc, and naftifine showed indifference (FICI > 0.5, no synergism); dihydroergotamine, ergoloid, telmisartan, azelastine, and doxazosin showed synergism (FICI = 0.375); and the compound nilotinib showed strong synergism (FICI = 0.1875) (Table 3). Interestingly, a previous study identified ketoconazole as a potential EPI for NorA (Abd El-Baky et al., 2019). This possible discrepancy in terms of synergy between ketoconazole and ciprofloxacin may be due to differences in the strains and methods used in this study.

**TABLE 3:** MIC of ciprofloxacin for SA1199B in combination with different compounds.

**Compound(s) with used concentration**	**MIC of ciprofloxacin in μg/mL (fold reduction)**	**FICI (fractional inhibitory concentration index)**
Ciprofloxacin	4 (0)	-
+ Dihydroergotamine (50 μM)	0.5 (8)	0.375
+ Ergoloid (25 μM)	0.5 (8)	0.375
+ Pimozide (3.125 μM)	2 (2)	0.75
+ Telmisartan (100 μM)	0.5 (8)	0.375
+ Ketoconazole (12.5 μM)	2 (2)	0.75
+ Maraviroc (100 μM)	2 (2)	0.75
+ Azelastine (100 μM)	0.5 (8)	0.375
+ Nilotinib (100 μM)	0.25 (16)	0.1875
+ Doxazosin (50 μM)	0.5 (8)	0.375
+ Naftifine (100 μM)	2 (2)	0.625
+ Reserpine (100 μM)	0.5 (8)	0.375

**The fold reduction of inhibitory activity in comparison to ciprofloxacin alone is indicated between brackets. All experiments were performed at least three times.**

Taken together our results, we found out that the MIC of ciprofloxacin was highly reduced in combination with nilotinib followed by the compounds dihydroergotamine, ergoloid, telmisartan, azelastine, and doxazosin.

### Visualization of the Putative Interaction of the Best NorA Inhibitors and the Substrate Ciprofloxacin With the NorA Homology Model Using PyMol

**FIGURE 1:**
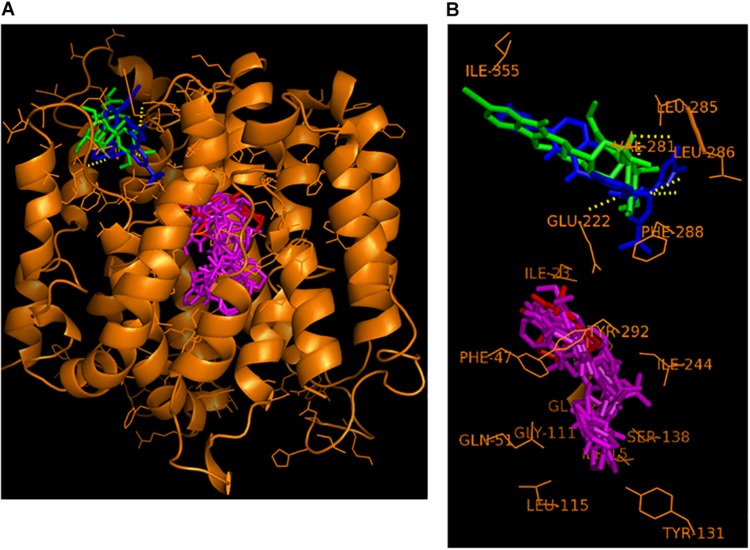
Hypothetical binding mode of nilotinib (in blue) and reserpine (in green) at the NorA groove binding site and of the substrate ciprofloxacin (in red) and the EPIs dihydroergotamine, ergoloid, azelastine, doxazosin, and telmisartan (all in magenta) at the NorA internal cavity binding site. **(A)** Overview of the entire NorA homology model with the bound compounds. **(B)** Close-up compound-residue interaction within a 3 Å radius.

The six best newly discovered NorA inhibitors that displayed synergism with ciprofloxacin (FICI of 0.375 or lower, Table 3) as well as the known NorA inhibitor reserpine were docked to the NorA homology model (including the substrate ciprofloxacin) using PyRx as described and the binding modes were visualized using PyMol. Although the docking was performed to the whole surface of the NorA homology model, only two distinctive binding sites, not embedded in the cytoplasmic membrane and hence freely accessible by substrates or EPIs, were discovered (Figures 1A,B). One binding site, delimited by the residues ILE-15, ILE-19, GLY-18, ILE-23, PHE-47, GLN-51, GLY-111, LEU-115, TYR-131, SER-138, ILE-244, and TYR-292, interacted both with the substrate ciprofloxacin as well as the NorA inhibitors dihydroergotamine, ergoloid, azelastine, doxazosin, and telmisartan. This region was referred to as “the internal cavity” in the publication describing the EmrD crystal structure, the coordinates of which were used for our NorA homology model. EmrD was used as template since it displays the highest sequence similarity to NorA (41%) of all crystalized bacterial MDR transporters belonging to the MFS (Yin et al., 2006).

In contrast, a second binding site that we name here as “the groove”, which is situated on top of the NorA protein and connected via a tiny channel with the internal cavity, was only populated by the two EPIs nilotinib and reserpine.

This alternative binding site, localized just outside of the transmembrane region embedded in the phospholipid bilayer of the cytoplasmic membrane, was delimitated by the residues VAL-281, LEU-285, LEU-286, PHE-288, GLU-222, and ILE-355.

### Efflux Assays

**FIGURE 2:**
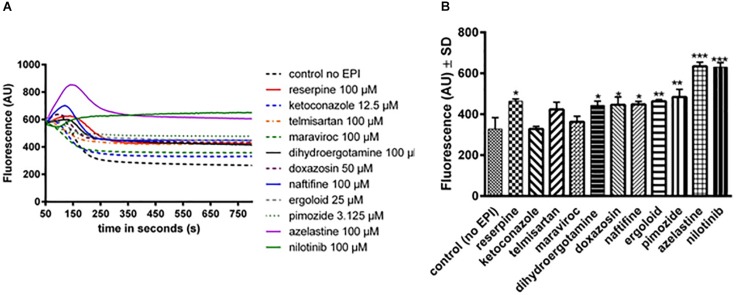
Assessment of efflux activity by a real-time fluorometric DiOC_3_ efflux assay in SA1199B with and without (= control no EPI) various compounds. **(A)** Real-time fluorometric assays were conducted in the presence of 56.25 mM glucose. Reserpine was used as control, as it is a known NorA efflux pump inhibitor. Each curve represents the mean of three independent experiments. **(B)** The fluorescence of the compounds at timepoint 350 s is shown. The bars and whiskers represent the means ± SD of three independent experiments. The differences between all of the compounds and control were analyzed by a one-way ANOVA test, with Dunnett’s multiple comparisons test (^∗^*p* < 0.05; ^∗∗^*p* < 0.01; and ^∗∗∗^*p* < 0.001).

A fluorescent efflux assay was used to measure the effect of the previously mentioned compounds on DiOC_3_ efflux in *S. aureus* SA1199B (*norA*+++). The fluorescent dye DiOC_3_ binds to the cytoplasmic side of bacterial membranes and its use in NorA efflux assays has been previously described (Zimmermann et al., 2017). Cells were resuspended in fresh phosphate potassium buffer (PPB) with and without the compounds and placed in the fluorimeter cuvette. After energization of the cells with glucose, a rapid decrease in fluorescence was observed. Compounds that inhibit the efflux of DiOC_3_ result in higher residual fluorescence, whereas non-inhibiting compounds or untreated cells extrude DiOC_3_, resulting in lower residual fluorescence. In the presence of various compounds, varying levels of efflux were observed (Figure 2A). The curves were normalized to 600 fluorescent arbitrary units (FAU) to allow better comparison. For a better overview, the fluorescence displayed at 350 s is shown in Figure 2B. This time point was chosen as the fluorescence levels remained relatively stable and the efflux capacities could be easily compared. Higher fluorescence levels indicate that more dye remains within the cells and that the inhibition of the NorA efflux pump is stronger (Figure 2B). The compounds ketoconazole, telmisartan, and maraviroc showed a nonsignificant inhibition of NorA (Figures 2A,B), whereas dihydroergotamine, doxazosin, naftifine, ergoloid, and pimozide showed a capacity to inhibit the efflux of DiOC_3_ that was similar to that of the positive control reserpine (Figures 2A,B). In addition, azelastine and nilotinib showed a very strong efflux pump inhibition (Figures 2A,B).

To further investigate the specific effect of nilotinib on NorA, the efflux of DiOC_3_ by this compound was tested for the strains *S. aureus* SA1199 (wild type) and the SAK1758 (Δ*norA*) in comparison to the strain SA1199B (*norA*+++) (Supplementary Figure S1). A lower but similar decrease in fluorescence was observed for the wild type strain while no effect was measured for the mutant strain (Supplementary Figure S1).

Collectively, our efflux assays suggest that nilotinib is a specific and strong efflux pump inhibitor of NorA.

### Determination of the Minimum Effective Concentration of Nilotinib in Combination With Ciprofloxacin

Because the compound nilotinib showed the highest FIC index value and the highest retardation of DiOC_3_ efflux, indicating that it is a good efflux pump inhibitor, we determined the minimum effective concentration of this compound. When nilotinib was added, the MIC of ciprofloxacin was reduced, with an effect observed at concentrations as low as 0.195 μM nilotinib (2-fold reduction of ciprofloxacin concentration for *S. aureus* SA1199 and SA1199B). No changes in the MIC were observed for the *S. aureus* SAK1758 (Δ*norA*) strain, indicating that nilotinib has a specific effect on the NorA efflux pump. The effects were more substantial in SA1199B, where a 4-fold reduction in ciprofloxacin at a nilotinib concentration of 0.78μM (Table 4), a concentration that is well achievable in human blood plasma (Tanaka et al., 2010).

**TABLE 4:** MIC of ciprofloxacin (μg/mL) in combination with different concentrations of nilotinib toward the strains SA1199 (wild type), SAK1758 (Δ*norA*) and SA1199B (*norA*+++).

	**Ciprofloxacin MIC (μg/mL)**				
	**MIC ciprofloxacin**	**+ 0.195 μM nilotinib**	**+ 0.39 μM nilotinib**	**+ 0.78 μM nilotinib**	**+ 1.56 μM nilotinib**
SA1199	0.25	0.125 (2)	0.125 (2)	0.125 (2)	0.125 (2)
SA1199B (*norA*+++)	4	2 (2)	2 (2)	1 (4)	1 (4)
SAK1758(*ΔnorA*)	0.016	0.016 (0)	0.016 (0)	0.016 (0)	0.016 (0)

**The fold reduction in the ciprofloxacin MIC in combination with nilotinib is indicated in brackets. Each value represents the mean of three independent experiments.**

### Biofilm Assays

According to the results of FICI and efflux assays, nilotinib was selected as the best inhibitor of NorA and follow-up experiments on biofilm were performed with the strain *S. aureus* SA1199B (*norA*+++). We defined the minimum concentration of a drug that can significantly inhibit biofilm formation as its BPC (Biofilm Prevention Concentration) and for eradication of a mature biofilm as BEC (Biofilm Eradication Concentration). Ciprofloxacin alone showed almost no effect on biofilms at concentrations of up to 8 μg/mL (BPC: Supplementary Figure S2A and BEC: Supplementary Figure S2B), as was observed in previous studies (Singh et al., 2010). Only at concentrations of 16 μg/mL and above did ciprofloxacin promote a decrease in biofilm biomass (Supplementary Figure S2). Nilotinib alone showed no inhibitory effect on biofilms as determined by measuring the BPC and BEC (Supplementary Figures S3A,B respectively).

**FIGURE 3:**
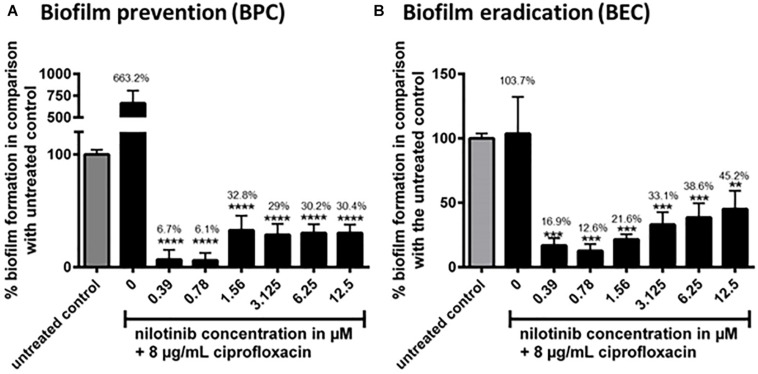
The effect of the combination of ciprofloxacin with nilotinib on biofilm prevention (BPC) and eradication (BEC). The bars and whiskers represent the means ± SD of three independent experiments. **(A)** Biofilm prevention concentration (BPC) of 8 μg/mL ciprofloxacin in combination with increasing concentrations of nilotinib. The value “0” indicates treatment with 8 μg/mL ciprofloxacin without the addition of nilotinib. **(B)** Biofilm eradication concentration of 8 μg/mL ciprofloxacin in combination with increasing concentrations of nilotinib. The value “0” indicates treatment with 8 μg/mL ciprofloxacin without the addition of nilotinib. The % values are shown on each column. The comparisons between untreated control and the serial dilutions of nilotinib were analyzed by one-way ANOVA with Dunnett’s multiple comparisons test (^∗∗^*p* < 0.01; ^∗∗∗^*p* < 0.001; and ^∗∗∗∗^*p* < 0.0001).

The BPC and BEC were screened with 8 μg/mL of ciprofloxacin (2 × MIC for *S. aureus* 1199B) in combination with serial dilutions of nilotinib (Figures 3A,B). The BPC showed significant inhibition of biofilm formation by treatment with 8 μg/mL of ciprofloxacin in combination with 0.39 μM of nilotinib. The BEC showed significant eradication of preformed biofilm by treatment with 8 μg/mL of ciprofloxacin in combination with 0.39 μM of nilotinib.

Similar results were obtained with the strain *S. aureus* SA1199 where *norA* is only expressed at a basal level by testing the BPC and BEC with 0.5 μg/mL of ciprofloxacin (2 × MIC for this strain) alone or in combination with serial dilutions of nilotinib (Supplementary Figures S4, S5).

**FIGURE 4:**
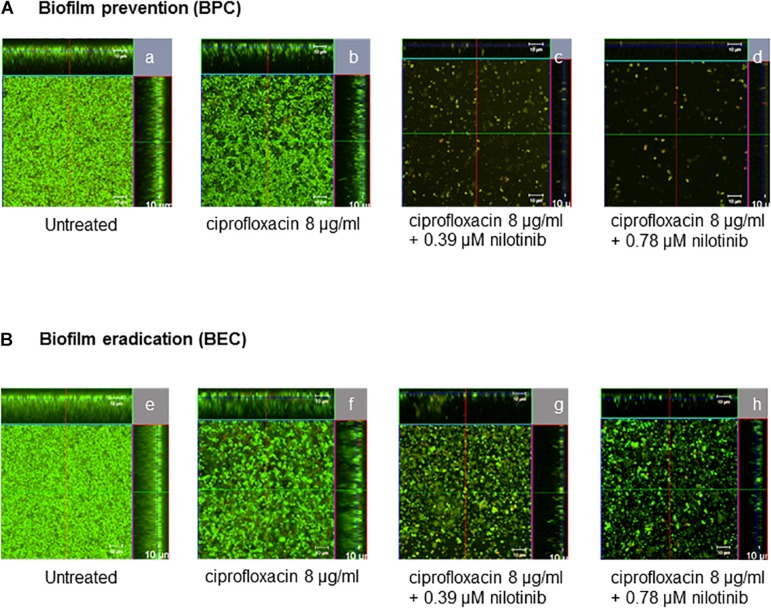
Confocal laser scanning microscopy ortho-images of LIVE/DEAD^®^-stained *S. aureus* SA1199B biofilms. The x/y planes correspond to the top views on basal biofilm layers and the marginal images corresponds to the cross-sections of the biofilms for **(A)** BPC and **(B)** BEC: (a,e) biofilms without treatment, (b,f) treatment with 8 μg/mL ciprofloxacin, (c,g) treatment with 8 μg/mL of ciprofloxacin in combination with 0.39 μM of nilotinib, and (d,h) treatment with 8 μg/mL ciprofloxacin in combination with 0.78 μM of nilotinib. Viable bacteria are visible in green and dead bacteria in red. The images are representative of three independent experiments.

**FIGURE 5:**
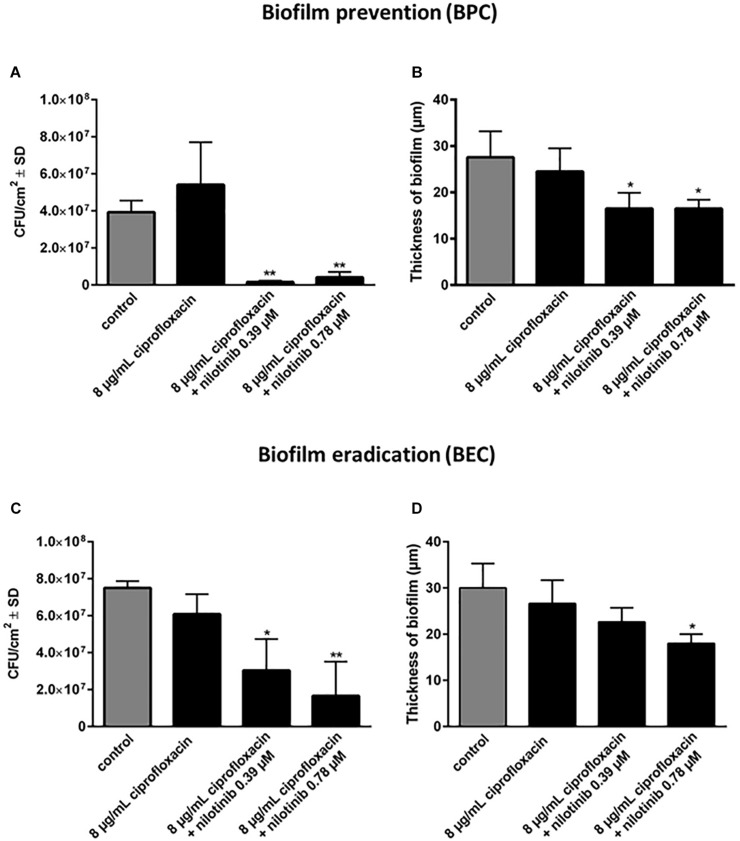
Quantification of the effects of nilotinib on biofilm formation (BPC) and mature biofilm (BEC) in combination with 8 μg/mL of ciprofloxacin. The viable bacterial cells (CFU/cm^2^) **(A,C)** and biofilm thickness **(B,D)** were determined using the qBA algorithm based on CLSM images (approximately 100 μm × 100 μm) and scaled up to an area of 1 cm^2^ (10^4^ cells/cm^2^ represents the limit of detection of this method). The experiments were performed three times and the means and standard deviations are shown. The comparison between untreated control and the different concentrations of nilotinib were analyzed by one-way ANOVA with Dunnett’s multiple comparisons test (^∗^*p* < 0.05 and ^∗∗^*p* < 0.01).

Additionally, confocal microscopic examination was used to investigate the effect of nilotinib and ciprofloxacin on biofilm structure (Figures 4, 5; Klinger-Strobel et al., 2016). Regions of green fluorescence (SYTO9) represent viable cells; the red fluorescence (propidium iodide) indicates non-viable cells (Figure 4). Untreated *S. aureus* SA1199B biofilms grown in the presence of TSB alone showed a majority of viable cells and a robust architecture (Figure 5). The viable cells and thickness of the biofilm were tested for BPC and BEC. The biofilm treated with ciprofloxacin in combination with nilotinib (0.39 and 0.78 μM) exhibited significant reduction on the viable cells for BPC and BEC in comparison to biofilm treated with ciprofloxacin alone (Figure 5A for BPC and Figure 5C for BEC). Moreover, biofilm treated with 8 μg/mL of ciprofloxacin and nilotinib (0.39 and 0.78 μM) showed a significant reduction of the thickness of the exopolymeric matrix and structural disruption in comparison to the untreated biofilm for BPC (60%; Figure 5B). However, the BEC only showed significant disruption of biofilm after the treatment with 8 μg/mL ciprofloxacin in combination with 0.78 μM of nilotinib (60%; Figure 5D). Thus, higher concentrations are required for eradication of biofilm because the compounds need to diffuse and penetrate into the already mature biofilm matrix. Furthermore, no effects of ciprofloxacin alone on viable bacteria or biofilm’s thickness were observed for the BPC and BEC (Figure 5).

Taken together our results showed an efficient prevention of biofilm formation and a significant reduction in pre-formed biofilm by the treatment with ciprofloxacin in combination with nilotinib.

### Toxicity Evaluation

To investigate a cytotoxic effect of nilotinib in the used concentrations on human cells, endothelial cells were incubated with this compound alone or in combination with 8 μg/mL of ciprofloxacin (Supplementary Figure S6). After 24 h, the cells stained with propidium iodide and cell death was measured by flow cytometry. The maximal cell death detected was 2% and no differences were observed between all treatments in comparison with the untreated control (Supplementary Figure S6). These results indicate that no possible cytotoxic effect is induced on endothelial cells by the treatment of nilotinib alone or in combination with ciprofloxacin.

## Discussion

Antibiotic resistance is a major problem for clinicians in their fight against bacterial infections. Important mechanisms of antibiotic resistance include antibiotic modification, antibiotic target alteration and antibiotic efflux by membrane transporters (Foster, 2017; Petchiappan and Chatterji, 2017). In recent years, several efflux pumps in gram-positive bacteria that extrude potentially harmful substances have been discovered and the NorA efflux pump is one of the most studied ones in *S. aureus* (Schindler and Kaatz, 2016). Given that it is overproduced in a considerable percentage of clinical *S. aureus* strains, the focus of research has been the investigation of potential EPIs. EPIs inhibit the extrusion of antibiotics, thus raising the intracellular concentration without raising the dose administered to the patient. As a result, formerly resistant bacteria may become sensitive again to a particular antibiotic substrate (Lomovskaya and Bostian, 2006; Costa et al., 2013). Many potential EPIs have been found, but unfortunately none of them is both clinically approved and reaches sufficient plasma levels (Lomovskaya and Bostian, 2006; Pletz et al., 2013). Thus, we set out to screen already approved drugs for potential efflux pump inhibition.

The initial screening was done by docking a virtual compound library of FDA-approved drugs to a NorA homology model. Although some inhibitors were discovered by using molecular docking, the predictive power was rather weak. For example, reserpine, a well-known inhibitor of the NorA efflux pump, was predicted to possess a free binding energy of -9.4 kcal/mol which suggests a much lower binding affinity than that of the predicted best binder (-13.2 kcal/mol, dihydroergotamine). A reason for this might be the putative inaccuracy of the homology model which used the efflux pump EmrD as a template. Although EmrD displays the highest sequence similarity of all crystalized bacterial MDR transporters belonging to the MFS (Yin et al., 2006), it is unclear whether our homology model represents an accurate model of NorA. Moreover, our model is based on an intermediate state of the EmrD crystal structure and not on an inwarding-facing or outward-facing conformation, typical of many MFS crystal structures.

Interestingly, in our docking model, two binding sites were discovered using whole protein docking.

One binding site on top of NorA, which we named “the groove,” interacted with nilotinib and the known EPI reserpine. The other binding site, designated as “the internal cavity,” bound the substrate ciprofloxacin and the EPIs dihydroergotamine, ergoloid, azelastine, doxazosin, and telmisartan, suggesting that these EPIs may be engaged in competitive inhibition of ciprofloxacin binding.

Although whole protein docking was used, both binding sites were found to be located outside of the transmembrane region of NorA that is embedded in the cytoplasmic membrane and should thus be freely accessible to the compounds. A known crystal structure of NorA would possibly produce more accurate results, but is currently not available. The four compounds with the highest binding energies did not show consistent synergistic activity with ciprofloxacin. Dihydroergotamine and ergoloid as well as telmisartan acted synergistically with ciprofloxacin (FICI = 0.375 for all three compounds) but pimozide did not (FICI = 0.75). Compounds with much lower binding energies also showed strong synergism (nilotinib, -10.8 kcal/mol with a FICI of 0.1875, and doxazosin, -9.4 kcal/mol with a FICI of 0.375). Thus, our results suggest a lack of correlation between synergistic activity and binding energy. This discrepancy might be due to the limitations of the NorA homology model based on the EmrD template, as discussed above. The next step was to test the ability substances to inhibit the extrusion of the fluorescent dye DiOC_3_ in efflux assays. Compounds with an effect similar to reserpine included dihydroergotamine, pimozide, ergoloid, and naftifine, while compounds with an efflux inhibitory effect better than reserpine included azelastine and nilotinib. The compound nilotinib showed a good synergism, with an FICI value of 0.1875 (<0.5), and it also maintained the fluorescence activity at a high level, suggesting that it inhibited dye extrusion, possibly via inhibition of the NorA efflux pump. Thus, we performed combination assays with nilotinib to determine the minimal effective concentration of nilotinib in combination with ciprofloxacin. Concentrations of nilotinib as low as 0.195 μM showed a twofold reduction in the MIC of ciprofloxacin. In contrast, there was no effect observed when nilotinib was administered in combination with ciprofloxacin to the *norA*-lacking strain (SAK1758) and only a small effect with the wild type strain (SA1199). Nilotinib itself is a substance used in the treatment of chronic myeloid leukemia where it acts by means of tyrosine kinase inhibition. A mean serum concentration as high as 2000 ng/mL [2 μg/mL (3.78 μM)] was reached in patients taking 400 mg doses of the drug (Tanaka et al., 2010). In this study, we observed an effect at one twentieth of the maximum concentration in combination with ciprofloxacin. This suggests nilotinib to be the first substance that can achieve specific efflux pump inhibition of NorA at clinically achievable concentrations. To test the clinical applications of nilotinib, we have further investigated its effect on biofilm formation. Bacterial biofilms are associated with a large number of infections that are difficult to treat. Bacteria may become tolerant to antibiotics due to a number of mechanisms such as a potential delay in penetration of antibiotics (e.g., decreased penetration of ciprofloxacin in *Pseudomonas aeruginosa* (Suci et al., 1994; Mah and O’Toole, 2001). Moreover, efflux pumps are upregulated during biofilm growth and established EPIs can significantly reduce preformed biofilm (Kvist et al., 2008). This makes efflux pumps attractive targets for anti-biofilm measures (Alav et al., 2018). Thus, we investigated the effect of nilotinib to prevent and eradicate biofilm. Intriguingly, rising concentrations of ciprofloxacin up to 8 μg/mL promote biofilm growth (Supplementary Figure S2A). This effect has been previously shown for *S. aureus* by other antibiotics, but to our knowledge not for ciprofloxacin (Hsu et al., 2011; Klinger-Strobel et al., 2016). An explanation might be increased stress which the bacteria are subjected to by the increasing antibiotic concentrations up to a point at which the antibiotic concentration becomes high enough to kill the bacteria (Alav et al., 2018). This elevated stress may lead to restructuring of the matrix, possibly favoring further biofilm growth due to the autolysis of some bacterial cells.

Biofilm inhibition was observed at levels far below the maximum plasma levels of nilotinib. The influence of the NorA efflux pump on biofilm formation is still largely unknown. Because nilotinib alone did not show an effect on biofilm formation, we presume that the effect of nilotinib on biofilms may not be due to a direct interruption of biofilm formation but rather due to inhibition of NorA, leading to an accumulation of ciprofloxacin. To the best of our knowledge, nilotinib is the first clinically approved drug that acts as efflux pump inhibitor against NorA and reaches plasma levels at which anti-biofilm activity occurs has been demonstrated in vitro. When administered locally (e.g., during osteomyelitis associated with biofilm formation), the dosage of nilotinib may possibly be raised, but further investigations are necessary to assess this possibility. Interestingly, nilotinib was both was observed to be effective in preventing biofilm formation and has an effect on already preformed biofilm. In cytotoxicity studies minimal cell death was observed, suggesting that the nilotinib and ciprofloxacin drug combination can be implemented in the clinic. However, further clinical trials are required to determine if the use of nilotinib is feasible for the reversal of bacterial MDR and for its anti-biofilm activity.

## Materials and Methods

### Bacterial Strains

*S. aureus* SA1199B is a known NorA efflux pump overproducer (*norA* + + +) (Kaatz et al., 1991). Strain SA1199 is the respective wildtype strain. Both bacterial strains were kindly provided by Glenn W. Kaatz (Detroit, MI, United States). *S. aureus* SAK1758 is a NorA-knockout strain and was kindly provided by Michael J. Rybak (Detroit, MI, United States).

### Chemicals

Ciprofloxacin was chosen as combination partner with the putative EPIs in the synergy tests as it is a substrate of the NorA pump (Fontaine et al., 2015). Ciprofloxacin was purchased from Sigma-Aldrich (Munich, Germany). The compounds were purchased from different merchants. Nilotinib and naftifine hydrochloride (naftifine) were obtained from Sigma-Aldrich (Munich, Germany). Dihydroergotoxine mesylate (ergoloid) and maraviroc were purchased from MedChem Express (Sollentuna, Sweden). Doxazosin mesylate (doxazosin), telmisartan, azelastine hydrochloride (azelastine), dihydroergotamine tartrate (dihydroergotamine), and ketoconazole were obtained from TCI Europe (Zwijndrecht, Belgium). Pimozide was purchased from Cayman Chemicals (Ann Arbor, MI, United States). Reserpine served as a control and was obtained from TCI Europe (Zwijndrecht, Belgium). The compounds were dissolved according to recommendations of the respective supplier.

### Molecular Docking

Since there is no published crystal structure of NorA, a homology model was created using the SWISSMODEL server and EmrD as a template (Yin et al., 2006; Waterhouse et al., 2018). EmrD, a homologous *Escherichia coli* efflux pump from the MFS (which NorA also belongs to), is available with its crystal structure at Protein Data Bank (Code: 2GFP). A virtual compound library of about 1200 FDA-approved drugs was used as ligands. Their modeled 3D structure coordinates were obtained from the e-Drug3D collection (freely available, e-Drug3D: 3D structure; Pihan et al., 2012).

### Virtual Screening

For molecular docking of the virtual compound library to the NorA homology model the programs PyRx version 0.8 and AutoDockVina were used (Trott and Olson, 2010; Dallakyan and Olson, 2015). The compounds were docked against the whole protein surface without any predefined binding sites and the results were sorted according to binding energy. PyMol (DeLano, 2002) was used as a visualization tool.

### Minimum Inhibitory Concentration Determination of Ciprofloxacin and Substances

This experiment was performed according to the microdilution protocol of CLSI (CLSI, 2015) using Mueller-Hinton broth (MHB; Carl Roth, Karlsruhe, Germany) and U-shaped 96-well plates (Greiner Bio One, Kremsmünster, Austria). The compounds were diluted in MHB in 2 mL tubes (Eppendorf, Wesseling-Berzdorf, Germany) to a concentration of 1.6 mM. Twelve twofold serial dilutions of the compounds were prepared in a 96-well plate to a final concentration ranging from 800 to 0.39 μM. The highest concentration was in well 12 of each row and the lowest concentration in well 1 of each row. Bacteria were grown overnight on Columbia blood agar plates and on the day of the experiment suspended in 0.9% saline solution with turbidity adjusted to a 0.5 McFarland standard. The bacterial solution was diluted in MHB to reach a bacterial concentration of 1.5 × 10^8^ CFU/mL and then added to the compounds in the wells to a final concentration of 5 × 10^5^ CFU/mL. After overnight incubation at 37°C the minimum inhibitory concentration (MIC) was determined by visual inspection to detect the lowest concentration of antimicrobial agent that completely inhibits growth of the organism in the tubes.

### Synergy Assays

Synergy assays of ciprofloxacin and compounds were performed according to the microdilution protocol of CLSI (CLSI, 2017) with slight modifications. Twelve twofold serial dilutions of ciprofloxacin were prepared with distilled and deionized water in a U-bottom 96-well plate such that the highest concentration was in column 12 (32 μg/mL) and the lowest was in column 1. After preparation, the 96-well plate was placed in an incubator overnight to evaporate the water in order to leave only the dried antibiotic substance. On the following day, the bacteria were prepared according to the Direct Colony Suspension Method of CLSI (CLSI, 2017) and diluted in MHB to a final concentration of 5 × 10^5^ CFU/mL. To test the synergistic activity between a compound and antibiotic, the compounds were added to the diluted bacteria to a final concentration of 1/4 × MIC but no higher than 100 μM. Compounds that did not show synergistic activity at 100 μM were regarded as not potent enough for putative clinical administration. Subsequently, 100 μL of the suspension was added to each well. The synergistic MIC (compound in combination with antibiotic) was determined by visual inspection as the first well with no visible turbidity. The observed MIC values were used to calculate the fractional inhibitory concentration index (FICI); this index allows evaluation of the combined effects of antibiotic and compound according to the following formula (Elion et al., 1954):

$$\mathrm{FICI}=\frac{\mathrm{MIC}\,\mathrm{of}\,\mathrm{antibiotic}\,\mathrm{and}\,\mathrm{compound}}{\mathrm{MIC}\,\mathrm{of}\,\mathrm{antibiotic}\,\mathrm{alone}}+\frac{\mathrm{MIC}\,\mathrm{of}\,\mathrm{antibiotic}\,\mathrm{and}\,\mathrm{compound}}{\mathrm{MIC}\,\mathrm{of}\,\mathrm{compound}\,\mathrm{alone}}$$

FICI was interpreted as follows: “synergy” – FICI less than or equal to 0.5; “indifference” – FICI greater than 0.5 and less than 4.0; “antagonism” – FICI greater than 4.0.

This experiment was repeated thrice (*n* = 3).

### NorA Efflux Assay

The NorA efflux assay was performed as described previously (Zimmermann et al., 2017). *S. aureus* strains were grown overnight at 37°C for 14–18 h with shaking (160 rpm) in LB broth (Luria/Miller, Carl Roth, Karlsruhe, Germany). To assess the effect of the compounds to inhibit ciprofloxacin efflux, the compounds were added to the resuspension buffer immediately before measurement. Bacteria were treated with 1/4 × MIC or 10 μM of each compound to avoid killing of the bacteria while still having a sufficient efflux pump inhibitory effect (described in section 4.6). This dose was chosen based on the use of compounds in the clinic, where a dosage greater than 100 μM cannot be safely administered.

### *In vitro* Combination Assay to Determine the Minimum Effective Concentration (MEC) of Nilotinib

Combination assays were performed similarly to the MIC testing in 96-well plates as described previously (Sy et al., 2016). To find out the MEC of nilotinib, we conducted checkerboard combination assays with different combinations of concentrations of this compound and ciprofloxacin. Each plate contained serial dilutions of the compound and ciprofloxacin in a checkerboard fashion (layout 10 × 8). Nilotinib was selected to be the best performing compound because it has the best FICI (best synergism with ciprofloxacin) and a high *in vitro* NorA efflux pump inhibition as determined by efflux assay.

Briefly, the final concentrations of nilotinib assayed ranged from 0.098 to 25 μM, and different concentrations ranges of ciprofloxacin (from 0.125 to 8 μg/mL for SA1199B; from 0.0078 to 0.5 μg/mL for SA1199 and SAK1758) due to the different susceptibilities of the strains to ciprofloxacin. Seven dilution steps for ciprofloxacin and 9 dilution steps for the nilotinib were analyzed. The plates were incubated at 37°C for 24 h. Each test was performed in triplicate and included a growth control where neither antibiotic nor compound was added. The MEC of nilotinib was determined as the concentration that produced at least a 2-fold reduction in the MIC of ciprofloxacin.

### Biofilm Assays

For biofilm formation overnight bacterial cultures were diluted 1:200 in fresh TSB (supplemented with 2.5% glucose). Subsequently, 200 μL of the diluted culture was added to each well of a flat-bottom 96-well plate and incubated with the lid on at 37°C for 48 h without shaking. The biofilm was stained with crystal violet, and the absorbance at 570 nm was measured as an indicator of the biofilm mass. The percentage of biofilm formation observed for each tested compound was calculated as described previously by using the formula (A_570_ - A_570_ of the untreated control) × 100 (Lopes et al., 2017). To confirm the results and to establish the live/dead ratio of the cells, the biofilms were assayed via confocal laser scanning microscopy with quantification by quantitative biofilm analysis (qBA) algorithm, as described previously (Klinger-Strobel et al., 2016).

#### Determination of the Biofilm Prevention Concentration (BPC)

To determine the concentration that can significantly inhibit biofilm formation (biofilm prevention concentration, BPC), nilotinib and ciprofloxacin were applied to a flat-bottom 96-well plate with lid (both Greiner Bio One, Kremsmünster, Austria) in the fashion of a checkerboard assay. The final concentrations of both compounds ranged from 0.39 to 100 μM for the compound nilotinib and from 0.5 to 32 μg/mL for the antibiotic ciprofloxacin for the strain SA1199B and from 0.03125 to 2 μg/mL ciprofloxacin for the strain SA1199. Dilutions were made with tryptic soy broth supplemented with 2.5% glucose. The bacteria were seeded into the wells at a 1:200 dilution and incubated at 37°C for 48 h without shaking.

For analysis of the biofilm formation the crystal violet staining method in a microtiter plate with modifications was applied (Merritt et al., 2005). Crystal violet stains the polysaccharide matrix (Peeters et al., 2008) and for the staining step, the supernatant was removed after 48 h and the plates were washed twice with PBS. Then 100 μL of the 1% crystal violet solution was added to the wells and incubated for 15 min at room temperature. The plates were washed three times with PBS and ethanol/acetone mixture (80:20) was added followed by incubation at room temperature for 10 min. Subsequently, the absorbance was measured at 570 nm with the microplate reader Infinite 200 Pro (Tecan, Männedorf, Switzerland). Wells incubated with medium alone served as a negative control and wells incubated with the bacterial strain SA1199B without any treatment served as a positive control.

#### Determination of the Biofilm Eradication Concentration (BEC)

To determine the concentration needed to significantly eradicate a mature biofilm (biofilm eradication concentration, BEC), the biofilm was grown as described above for 48 h without any compounds or antibiotics in tryptic soy broth supplemented with 2.5% glucose. After 48 h the liquid was removed and the wells were washed once with 200 μL PBS. Nilotinib and ciprofloxacin were added in the fashion of a checkerboard assay. The final concentrations ranged from 0.39 to 100 μM for the compound nilotinib and from 0.5 to 32 μg/mL for ciprofloxacin for the strain SA1199B and from 0.03125 to 2 μg/mL ciprofloxacin for the strain SA1199, dilutions were made with tryptic soy broth. After 24 h the biofilm was washed with 200 μL of PBS and stained with crystal violet as described above [section Determination of the biofilm prevention concentration (BPC)].

#### Biofilm Imaging and Computed Analysis

Biofilms were stained with the LIVE/DEAD BacLight Bacterial Viability Kit for microscopy (Life Technologies GmbH, Darmstadt, Germany) according to the manufacturer’s protocol (Thieme et al., 2018). SYTO9 stains the nucleic acid of living and dead cells and propidium iodide stains only the dead cells (Peeters et al., 2008). Stained biofilms were analyzed under vital conditions using an inverse confocal laser scanning microscope (CLSM) LSM780 with a 40 × air objective (Carl Zeiss AG, Jena, Germany) at 490 nm excitation by an argon laser. An area of ca. 100 μm (x-axis) × 100 μm (y-axis) was screened in 2 μm intervals in the z-axis (z-stack) in the green (emission 522 nm) and red (emission 635 nm) channels, respectively. The biofilm images were visualized by ZEN 9.0 software (Carl Zeiss AG, Jena, Germany). The biofilm experiments [eradication (BEC) and prevention (BPC)] were independently performed in triplicate for each assay. Quantitative analysis of biofilm images was performed by an algorithm termed qBA (quantitative biofilm analysis) that determined the number of bacterial counts/cm^2^ (Klinger-Strobel et al., 2016; Thieme et al., 2018).

### Test of Cytotoxicity on Endothelial Cells

The endothelial cells (EA.hy926 (ATCC^®^ CRL-2922^TM^) were grown on a 12-well plate until 80% of confluence. The cells were incubated with nilotinib alone (0.39, 0.78, and 1.56 μM), 8 μg/mL ciprofloxacin alone, or 8 μg/mL ciprofloxacin in combination with each of the aforementioned three nilotinib concentrations for 24 h at 37°C and 5% CO_2_. After the incubation time, the cell death was measured by flow cytometry. The rate of cell death was determined by measuring the uptake of propidium iodide (PI) as described previously (Nicoletti et al., 1991; Haslinger et al., 2003).

### Statistical Analysis

The differences between the effects of all compounds and ciprofloxacin alone were determined using GraphPad Prism version 4.00 (Graphpad, La Jolla, CA, United States). The normality of the distribution was analyzed with the D’Agostino & Pearson omnibus, and the Shapiro–Wilk normality test. Multiple groups were compared by an ordinary one-way ANOVA test, followed by Dunnett’s multiple comparisons test. According to the p-values, the differences were: either not significant (ns, *p* > 0.05); or significant (^∗^*p* < 0.05; ^∗∗^*p* < 0.01; ^∗∗∗^*p* < 0.001; and ^∗∗∗∗^*p* < 0.0001).

## Data Availability Statement

All datasets generated for this study are included in the article/Supplementary Material.

## Author Contributions

SZ performed all the experiments, interpretation of data for the work, and wrote the manuscript. MK-S performed confocal analysis and wrote sections of the manuscript. JB performed the docking analysis, interpretation of data, contributed the conception of the work, and wrote sections of the manuscript. SW performed all the cell cytotoxicity experiments. JR contributed to the conception of the work. MP and BL provided approval for publication of the content and contributed to write the manuscript. LT conceived and designed of the study, performed the statistical analysis, and wrote the manuscript. All authors contributed to manuscript revision, read and approved the submitted version.

## Conflict of Interest

The authors declare that the research was conducted in the absence of any commercial or financial relationships that could be construed as a potential conflict of interest.
