# Supplementary material for: Clinically Approved Drugs Inhibit the Staphylococcus aureus Multidrug NorA Efflux Pump and Reduce Biofilm Formation
Source: Front Microbiol. 2019 Dec 3;10:2762. doi: 10.3389/fmicb.2019.02762 (PMC6901667; doi:10.3389/fmicb.2019.02762)
Supplement: Supplementary file 1 [file Presentation_1.pptx]

## Slide 1
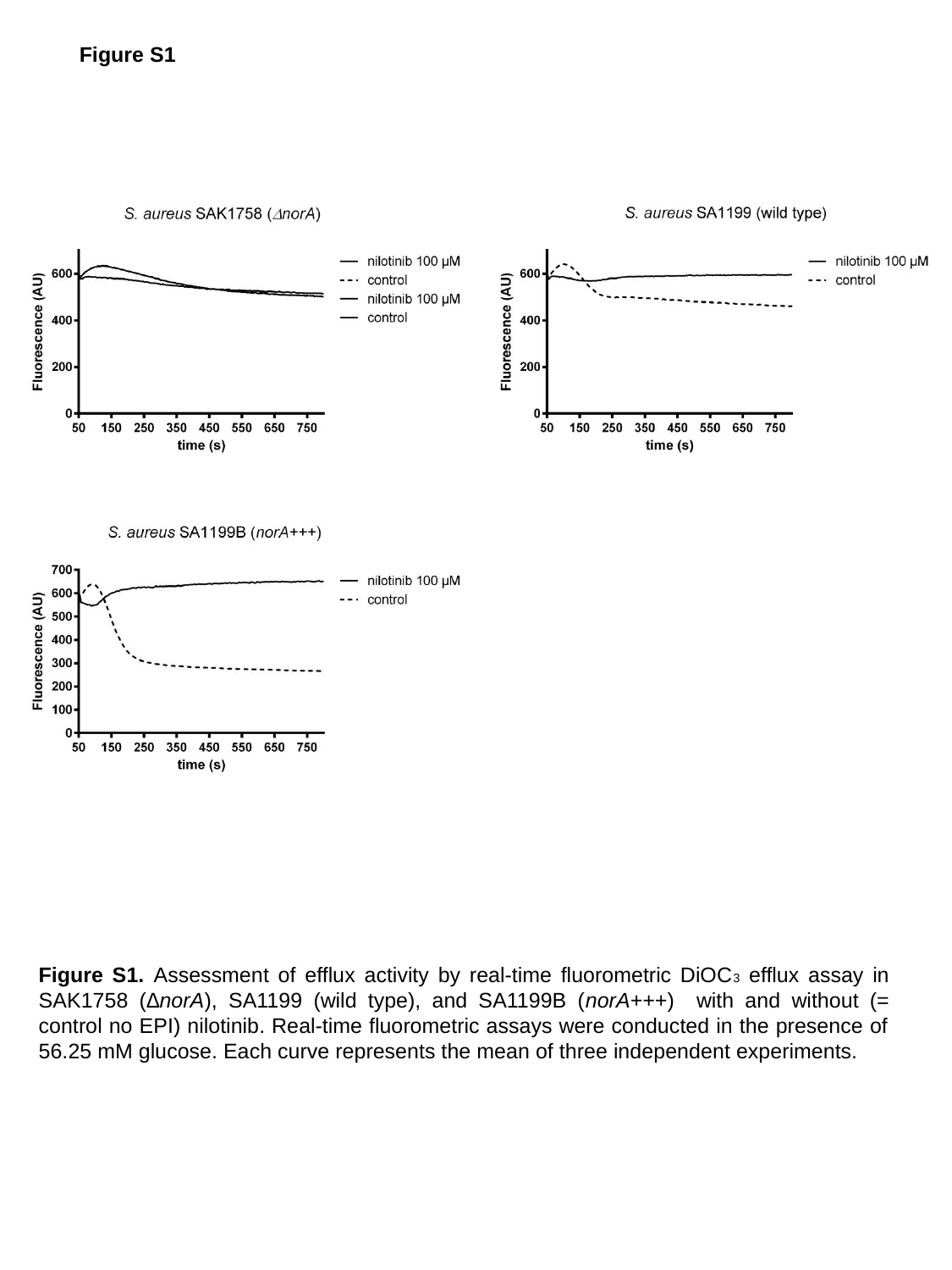

Figure S1
Figure S1. Assessment of efflux activity by real-time fluorometric DiOC3 efflux assay in SAK1758 (∆norA), SA1199 (wild type), and SA1199B (norA+++) with and without (= control no EPI) nilotinib. Real-time fluorometric assays were conducted in the presence of 56.25 mM glucose. Each curve represents the mean of three independent experiments.

## Slide 2
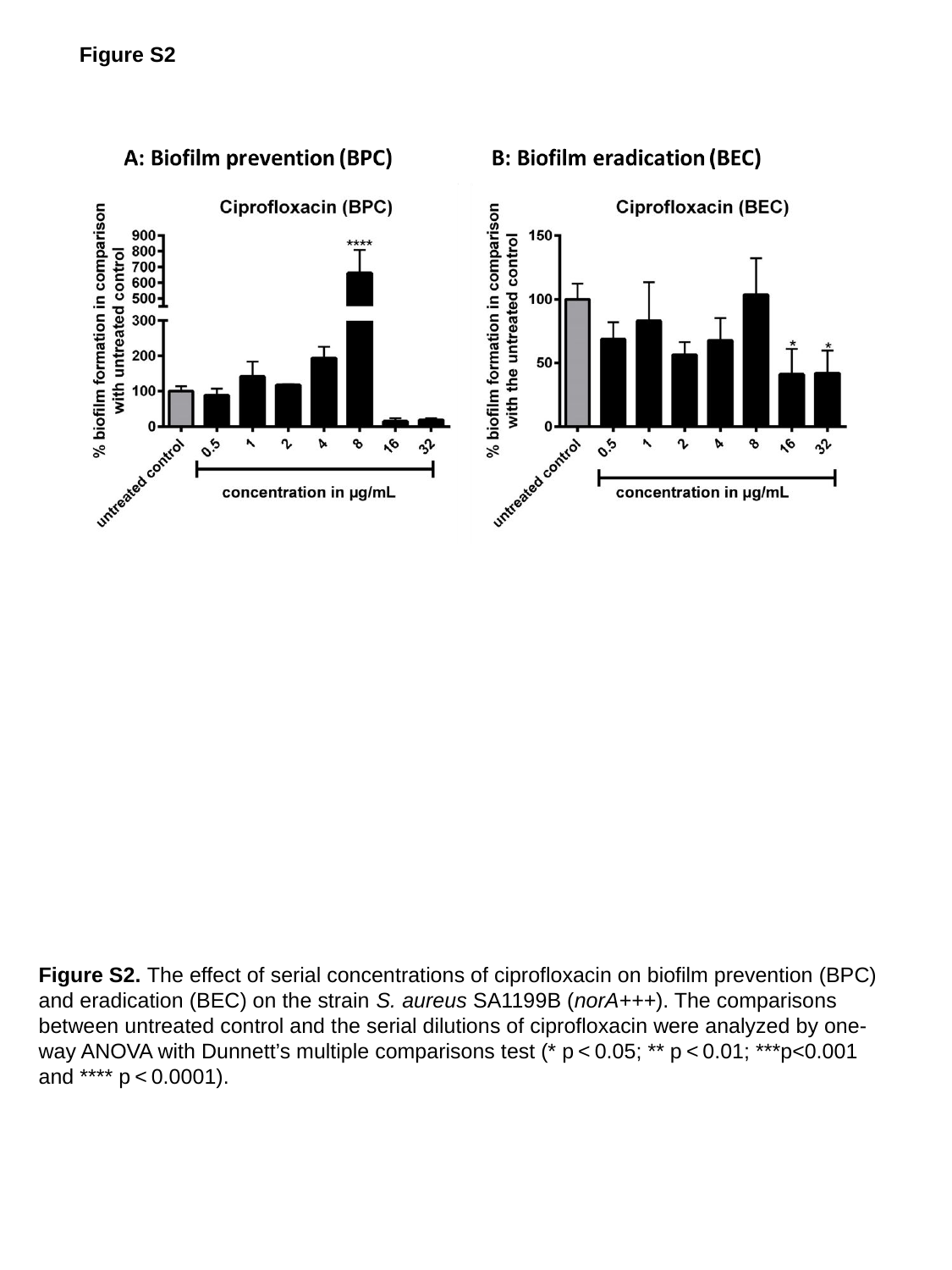

Figure S2
Figure S2. The effect of serial concentrations of ciprofloxacin on biofilm prevention (BPC) and eradication (BEC) on the strain S. aureus SA1199B (norA+++). The comparisons between untreated control and the serial dilutions of ciprofloxacin were analyzed by one-way ANOVA with Dunnett’s multiple comparisons test (* p < 0.05; ** p < 0.01; ***p<0.001 and **** p < 0.0001).

## Slide 3
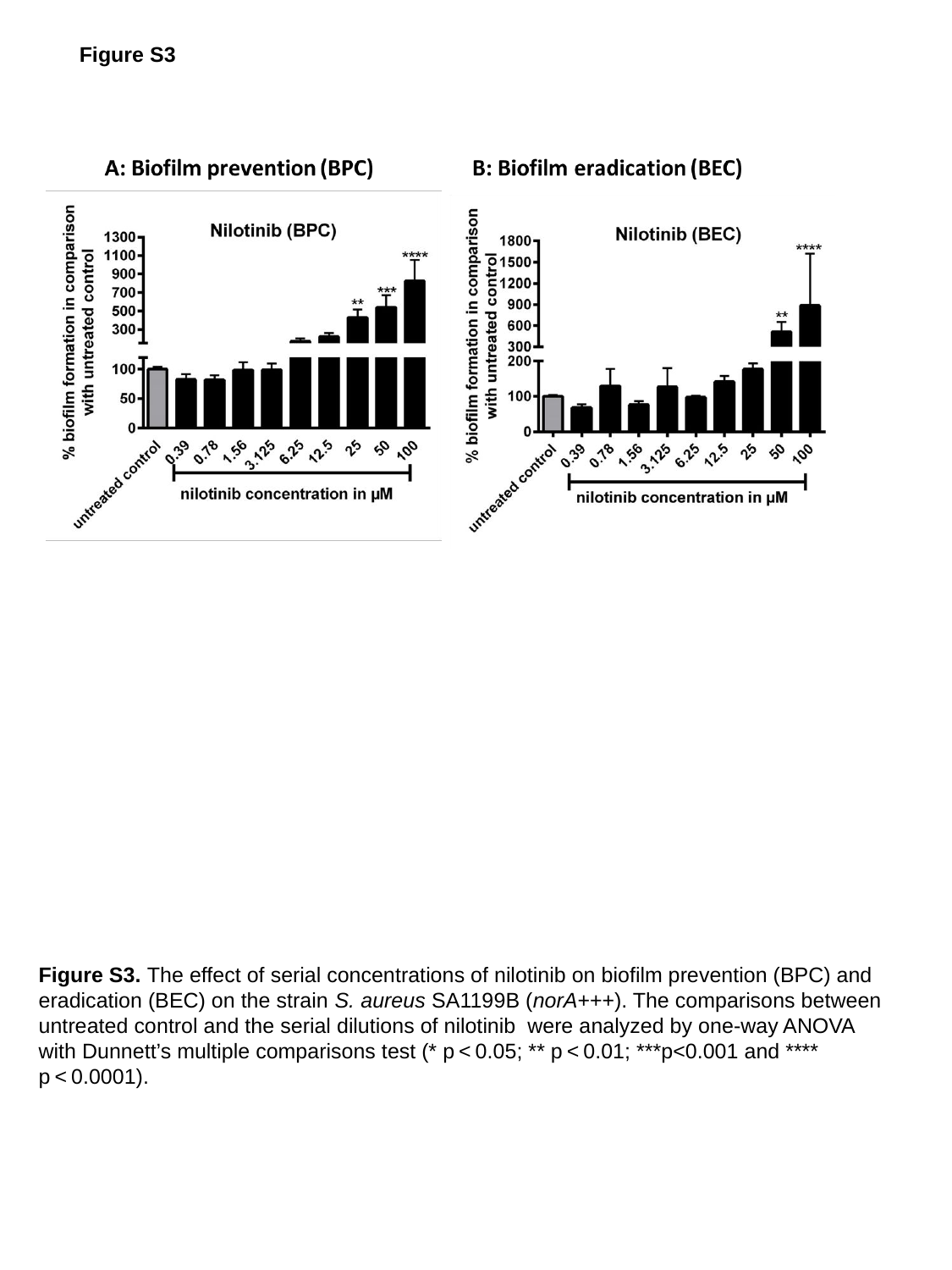

Figure S3
Figure S3. The effect of serial concentrations of nilotinib on biofilm prevention (BPC) and eradication (BEC) on the strain S. aureus SA1199B (norA+++). The comparisons between untreated control and the serial dilutions of nilotinib were analyzed by one-way ANOVA with Dunnett’s multiple comparisons test (* p < 0.05; ** p < 0.01; ***p<0.001 and **** p < 0.0001).

## Slide 4
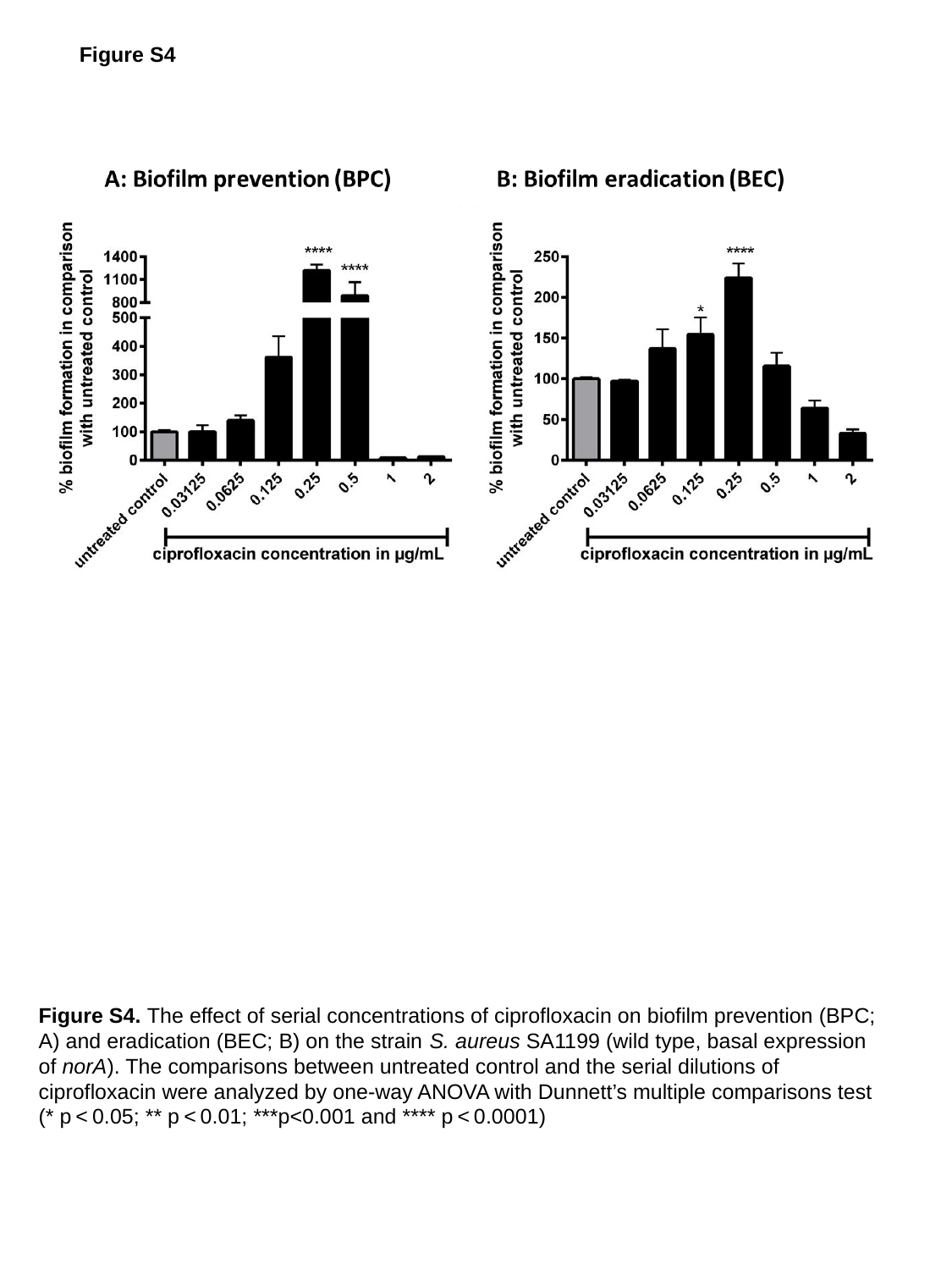

Figure S4
Figure S4. The effect of serial concentrations of ciprofloxacin on biofilm prevention (BPC; A) and eradication (BEC; B) on the strain S. aureus SA1199 (wild type, basal expression of norA). The comparisons between untreated control and the serial dilutions of ciprofloxacin were analyzed by one-way ANOVA with Dunnett’s multiple comparisons test (* p < 0.05; ** p < 0.01; ***p<0.001 and **** p < 0.0001)

## Slide 5
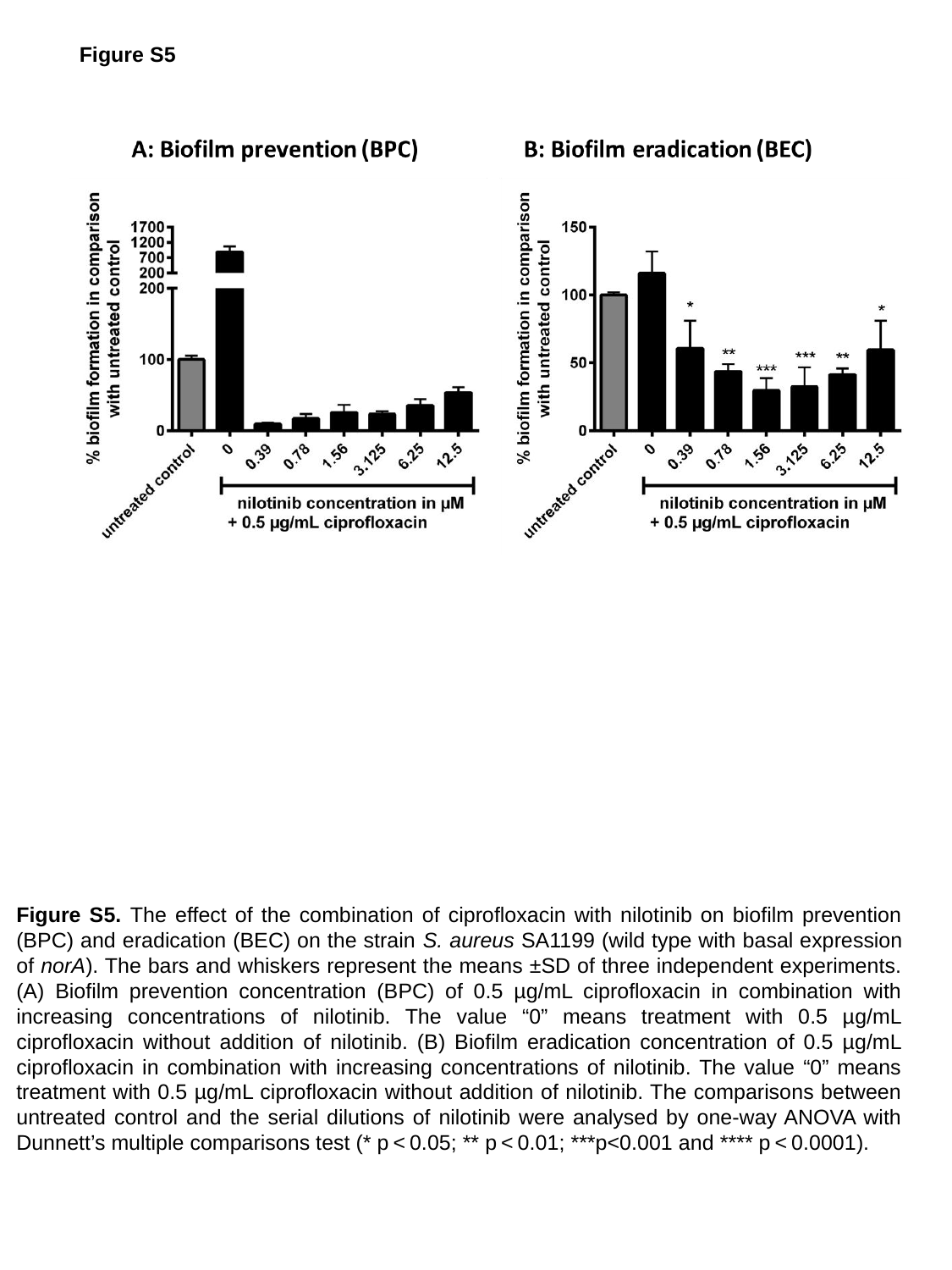

Figure S5
Figure S5. The effect of the combination of ciprofloxacin with nilotinib on biofilm prevention (BPC) and eradication (BEC) on the strain S. aureus SA1199 (wild type with basal expression of norA). The bars and whiskers represent the means ±SD of three independent experiments. (A) Biofilm prevention concentration (BPC) of 0.5 µg/mL ciprofloxacin in combination with increasing concentrations of nilotinib. The value “0” means treatment with 0.5 µg/mL ciprofloxacin without addition of nilotinib. (B) Biofilm eradication concentration of 0.5 µg/mL ciprofloxacin in combination with increasing concentrations of nilotinib. The value “0” means treatment with 0.5 µg/mL ciprofloxacin without addition of nilotinib. The comparisons between untreated control and the serial dilutions of nilotinib were analysed by one-way ANOVA with Dunnett’s multiple comparisons test (* p < 0.05; ** p < 0.01; ***p<0.001 and **** p < 0.0001).

## Slide 6
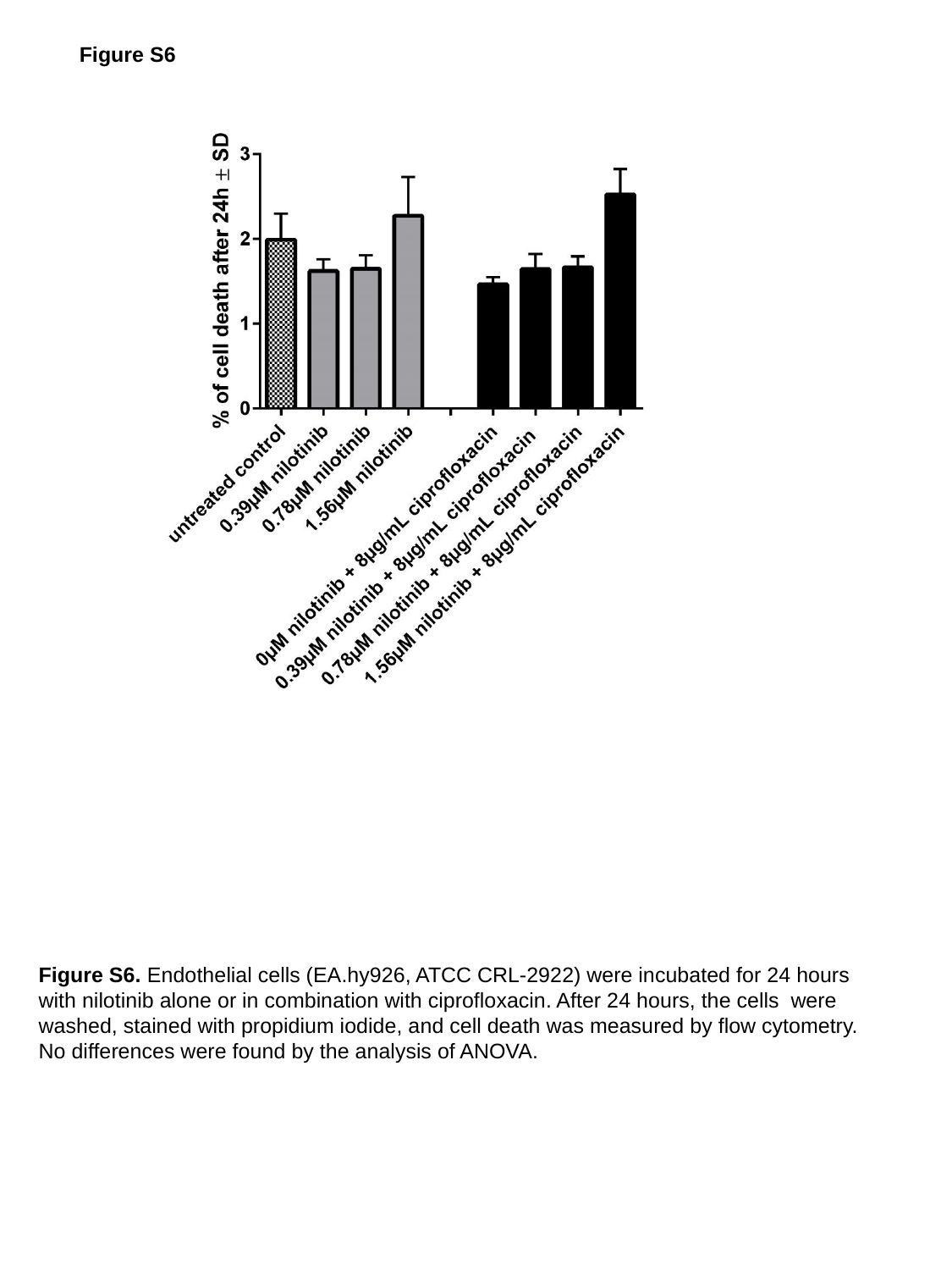

Figure S6
Figure S6. Endothelial cells (EA.hy926, ATCC CRL-2922) were incubated for 24 hours with nilotinib alone or in combination with ciprofloxacin. After 24 hours, the cells were washed, stained with propidium iodide, and cell death was measured by flow cytometry. No differences were found by the analysis of ANOVA.
